# Reconstruction of the Functional Ecosystem in the High Light, Low Temperature Union Glacier Region, Antarctica

**DOI:** 10.3389/fmicb.2019.02408

**Published:** 2019-10-18

**Authors:** Yi Li, Qian-Qian Cha, Yan-Ru Dang, Xiu-Lan Chen, Min Wang, Andrew McMinn, Giannina Espina, Yu-Zhong Zhang, Jenny M. Blamey, Qi-Long Qin

**Affiliations:** ^1^State Key Laboratory of Microbial Technology, Marine Biotechnology Research Center, Shandong University, Qingdao, China; ^2^Laboratory for Marine Biology and Biotechnology, Qingdao National Laboratory for Marine Science and Technology, Qingdao, China; ^3^College of Marine Life Sciences, Institute for Advanced Ocean Study, Ocean University of China, Qingdao, China; ^4^Institute for Marine and Antarctic Studies, University of Tasmania, Hobart, TAS, Australia; ^5^Fundación Científica y Cultural Biociencia, Santiago, Chile; ^6^Faculty of Chemistry and Biology, Universidad de Santiago de Chile, Santiago, Chile

**Keywords:** microorganism community, biogeochemical cycling, extreme environmental adaption, metagenomic analysis, antarctic glacier

## Abstract

Antarctica is covered by multiple larger glaciers with diverse extreme conditions. Microorganisms in Antarctic regions are primarily responsible for diverse biogeochemical processes. The identity and functionality of microorganisms from polar glaciers are defined. However, little is known about microbial communities from the high elevation glaciers. The Union Glacier, located in the inland of West Antarctica at 79°S, is a challenging environment for life to survive due to the high irradiance and low temperatures. Here, soil and rock samples were obtained from three high mountains (Rossman Cove, Charles Peak, and Elephant Head) adjacent to the Union Glacier. Using metagenomic analyses, the functional microbial ecosystem was analyzed through the reconstruction of carbon, nitrogen and sulfur metabolic pathways. A low biomass but diverse microbial community was found. Although archaea were detected, bacteria were dominant. Taxa responsible for carbon fixation were comprised of photoautotrophs (Cyanobacteria) and chemoautotrophs (mainly Alphaproteobacterial clades: *Bradyrhizobium*, *Sphingopyxis*, and *Nitrobacter*). The main nitrogen fixation taxa were *Halothece* (Cyanobacteria), *Methyloversatilis*, and *Leptothrix* (Betaproteobacteria). Diverse sulfide-oxidizing and sulfate-reducing bacteria, fermenters, denitrifying microbes, methanogens, and methane oxidizers were also found. Putative producers provide organic carbon and nitrogen for the growth of other heterotrophic microbes. In the biogeochemical pathways, assimilation and mineralization of organic compounds were the dominant processes. Besides, a range of metabolic pathways and genes related to high irradiance, low temperature and other stress adaptations were detected, which indicate that the microbial communities had adapted to and could survive in this harsh environment. These results provide a detailed perspective of the microbial functional ecology of the Union Glacier area and improve our understanding of linkages between microbial communities and biogeochemical cycling in high Antarctic ecosystems.

## Introduction

The Union Glacier, located in the Ellsworth Mountains of West Antarctica inland, is a large and heavily crevassed glacier (Cordero et al., 2014). Due to the high altitude (around 700 m), low aerosols and high surface albedo, it experiences very high irradiances. It also experiences seasonally high incident ultraviolet (UV) radiation due to seasonal ozone losses (Flemming et al., 2010). The ambient temperature between April and September is around −26 to −28°C and around −6°C in January (Cordero et al., 2014; Rivera et al., 2014). Under the extreme freezing condition, there is almost no liquid-water with most snowfall losses due to direct sublimation. Although climate warming has had a substantial impact on polar regions elsewhere (e.g., the Antarctic Peninsula and West Antarctica) and the cryosphere is rapidly shrinking (Rivera et al., 2010; Boetius et al., 2015), the Union Glacier has remained stable and still has a thickness of 1450 m without any obvious ice velocity changes in recent decades (Rivera et al., 2010, 2014).

Most previous studies of polar environments have focused on the less extreme sub-Antarctic and maritime Antarctic Peninsula locations, which have fundamentally different biomes (Chan et al., 2013). Aquatic microorganisms, for instance, have been found in diverse habitats including Antarctic lakes, ponds and coastal fringes (Chan et al., 2013; Cavicchioli, 2015; Llorens-Marès et al., 2015). In the hyper-arid polar desert of the Dry Valleys, functional traits of specialized microbial communities from distinct lithic and soil niches have been identified (Pointing et al., 2009; Chan et al., 2013). Glaciers and ice sheets, which make up a significant proportion of the earth’s cryosphere, contain unique biomes inhabited by microbial communities of all three domains of life (Boetius et al., 2015; Garcia-Lopez and Cid, 2017). High biodiversities of prokaryotes and eukaryotes from other terrestrial glaciers have already been found (Varin et al., 2012; Garcia-Lopez and Cid, 2017). In addition, the identity and functional capacity of microorganisms from polar coastal glaciers have been defined (Boetius et al., 2015). However, little is known about microbial communities from the high elevation glaciers such as the Union Glacier and almost nothing is known about the stress tolerance mechanisms for climatic extremes in edaphic Antarctic microorganisms (Chan et al., 2013; Barahona et al., 2016). Metagenomic analyses, through the assembly of phylogenetic and biological functional inventories, have made studies in microbial community structure, metabolic potential and ecology (e.g., carbon, nitrogen and sulfur cycling) more straightforward and accurate and allow *in situ* ecological processes and microbial interactions to be inferred (Lauro et al., 2011; Prosser, 2012; Li et al., 2018).

Here, using a combination of both 16S rRNA gene amplicon and direct metagenomic analyses, the soil microorganisms from mountain surroundings of the Union Glacier were explored. After phylogenetic and functional identification, linkages between microbial community structures and ecological processes for carbon, nitrogen and sulfur cycling were studied in detail. The relative abundance and distribution of marker genes acted as an agent of the potential *in situ* biogeochemical metabolic pathways. Although the glacier ecosystem experiences extreme climatic conditions, which are usually considered to be hostile to life (Cordero et al., 2014), several metabolic pathways and genes related to stress resistance were analyzed and diverse microbial communities could colonize. Here we reconstruct the ecosystem of the Union Glacier to explore the metabolic potential with respect to carbon, nitrogen and sulfur biogeochemical cycling.

## Materials and Methods

### Location Description and Sample Collection

**FIGURE 1:**
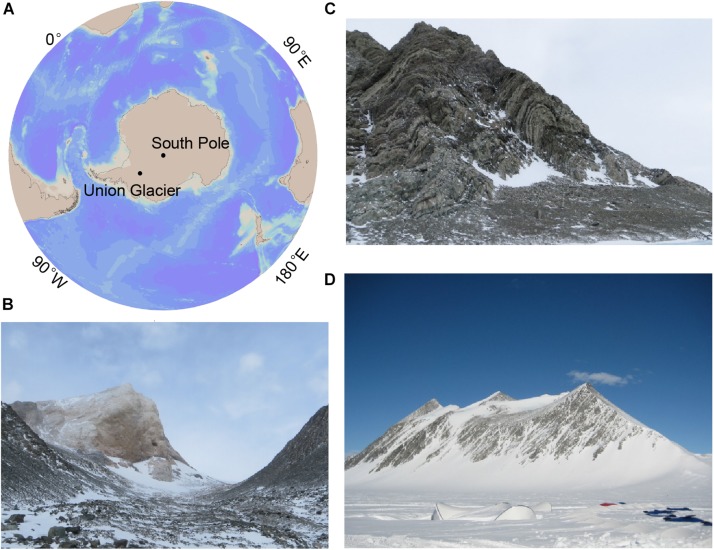
Union Glacier site and photos of sampled glacier mountains. **(A)** Map of Antarctica illustrating the location of Union Glacier. **(B)** Photograph of the Elephant Head. **(C)** Photograph of the Charles Peak. **(D)** Photograph of the Rossman Cove.

Sample sites were located around the Union Glacier (79°45′S, 82°30′W), Antarctica (Figure 1A). A total of 6 samples were obtained from three mountains (namely Rossman Cove, Charles Peak and Elephant Head) during Antarctic summer (Figures 1B–D). Sampling locations, sample descriptions and chemical parameters are given in Table 1. Samples were classified into 4 groups based on their locations and layers, namely CP, EH, GU_S, and GU_D, respectively. All soil and rock samples were stored in sterile tubes (Corning Inc., United States) and then instantly frozen and stored at −80°C (Thermo Fisher Scientific, United States) in the lab until nucleic acid extraction.

**TABLE 1:** Summary of the environmental samples from the Union Glacier area.

**Sample**	**GPS**	**Location**	**Description**	**Depth**	**pH**	**Temperature**
GU7	79° 47.51′ S/ 82° 59.14′ W	Rossman Cove	Soils obtained at 5 cm depth from Rossman Cove summit	Subsurface	6	−4.3°C
GU8	79° 47.51′ S/ 82° 59.14′ W	Rossman Cove	Soils from Rossman Cove summit	Surface	4.5	−9.8°C
CP14	79° 43.85′ S/ 83° 11.94′ W	Charles Peak	Soils from Charles Peak summit	Surface	6	−9.1°C
EH2	79°49.30′ S/ 83°20.42′ W	Elephant Head	Disagregate white rock from Elephant Head moraine	Surface	6	−8°C
EH5	79°49.30′ S/ 83°20.42′ W	Elephant Head	Brown soils from Elephant Head moraine	Surface	6	−8°C
EH7	79°49.30′ S/ 83°20.42′ W	Elephant Head	Soils obtained from the “eye” surface of the Elephant head	Surface	6	−20°C

### DNA Extraction and Real-Time Quantitative PCR

Total genomic DNA from each sample was extracted with a PowerSoil^®^ DNA Isolation Kit (MOBIO, United States). The purity and concentration of extracted DNA were checked by agarose gel electrophoresis and micro-spectrophotometer (Nano-100), respectively. Then DNA was prepared for real-time quantitative PCR (qPCR), 16S rRNA gene amplicon and metagenomic sequencing. The qPCR amplifications were performed using LightCycler^®^ 480 II (Roche, Switzerland) following the instruction of SYBR^®^ Premix Ex TaqTM (Takara, Japan). Archaeal 16S rRNA genes were quantified using the primer Arc915R (5′-GTGCTCCCCCGCCAATTCCT-3′) and Arc344F (5′-ACGGGGYGCAGCAGGCGCGA-3′). Analogously, bacterial 16S rRNA genes were quantified using the primer Bac338F (5′-ACTCCTACGGGAGGCAGCA-3′) and Bac806R (5′-GGACTACHVGGGTWTCTAAT-3′) (Li et al., 2018). All standard curves were conducted using a dilution series (between 1 × 10^2^ and 1 × 10^7^ copies/μL) of a known amount of plasmids with cloned partial 16S rRNA genes separately for archaea and bacteria.

### 16S rRNA Gene Amplicon, Statistical, and Co-occurrence Analysis

Due to low biomass, Archaeal 16S rRNA gene sequencing could not be implemented. Bacterial 16S rRNA genes were amplified using barcoded sequencing primers 338F and 806R. The pooled PCR products were prepared as Illumina MiSeq paired-end libraries and sequenced (Majorbio Bio-Pharm Technology Co., Ltd., China). The obtained raw reads were trimmed to remove primer sequences and barcode sequences using Trimmomatic (Bolger et al., 2014). The trimmed reads were processed following the pipeline of Usearch (Edgar, 2018). Pair-wise reads were merged, then sequence reads from 6 samples were pooled and filtered with a maximum expected error threshold. After standard quality control, remaining reads were assigned to each OTU using the UPARSE-OTU algorithm with a 97% identity (Edgar, 2013). Meanwhile, Chimeric sequences were detected and removed. Obtained reads finally were used to produce an OTUs table. All taxonomic assignments were based on representative OTU sequences. These representative sequences were aligned and annotated through RDP Naive Bayesian rRNA Classifier at an 80% confidence threshold (Wang et al., 2007). The similarity threshold of OTUs assigned to phylum and genera were 100 and 50%, respectively.

Datasets of the OTUs table were normalized for alpha and beta diversity comparisons through the pipeline of qiime 2 (Hall and Beiko, 2018). The OTUs table was transformed into relative abundances for NMDS (non-metric multidimensional scaling) analyses by Primer 6 (Plymouth Marine Laboratory, United Kingdom). Bacterial community compositions and comparisons to relative abundances in different samples were performed by R software. Additionally, potential interactions between microbes were investigated through modeling community structure networks. The correlation network pattern was performed by python and was visualized by Gephi (Bastian et al., 2009). For a valid network analysis, a SparCC correlation coefficient >|0.65| was calculated with statistically significance (*P* < 0.01) (Barberán et al., 2012).

### Metagenomic Assembly and Functional Assignment

Due to insufficient sample size at some sites, metagenomic sequencing was only completed on EH7 soils. After removal of joint sequences by SeqPrep, raw Illumina reads with a length less than 50 bp, with an average base quality of less than 20 and those with ambiguous base “N” were filtered out using Sickle^1^. Screened paired-end reads were assembled using the IDBA-UD (Peng et al., 2011) algorithm with a kmer size iterated from 67 to 97. Based on the number of contigs (>500 bp), N50/N80 values, longest contig and the total length of the assembly, the kmer size 87 was selected for all further analysis (White et al., 2015). The set of open reading frames (ORFs) was predicted and each ORF was translated into amino acid sequences by Prodigal (Hyatt et al., 2010). Predicted genes were functionally and taxonomically annotated using GhostKOALA (Kanehisa et al., 2016). Functional categorizations of genes were identified through orthology assignment by eggNOG-mapper (Huerta-Cepas et al., 2017). In the metagenomic data, raw reads were matched back to the predicted gene set by Salmon to calculate the abundance of each gene (Patro et al., 2017).

As previously reported (Lauro et al., 2011), in this study carbon, nitrogen and sulfur marker genes were inferred through KEGG pathways and BRITE hierarchies with a few modifications (Supplementary Table S1). Also, the taxonomic identity assigned to each functional gene was determined. The genetic potential of microbial communities in biogeochemical cycles was assessed using a combination of marker genes. Each gene combination (the sum of multiple enzyme abundances in the same conversion step) was summed, which consisted of single or multiple metabolic subprocesses. Different combinations were comparatively analyzed among C, N, and S cycling as agents of the potential *in situ* relevance of these pathways. Because the marker genes *aprA* and *dsrA* could both mediate sulfide oxidation and dissimilatory sulfate reduction, they were assigned to the sulfate reduction or sulfide oxidation step according to phylogeny, i.e., they had a best match to an ortholog from a sulfate-reducing or sulfur-oxidizing clade (Llorens-Marès et al., 2015). Additionally, the functional analyses focused on environmental adaption to conditions found in the vicinity of the glacier (Supplementary Tables S2, S3). Bar-plots and heat-plots were generated using the library “gplots” of R software.

### Data Availability Statement

Trimmed 16S rRNA gene reads of all 6 samples and the raw metagenomic reads of EH7 have been deposited in the NCBI Sequence Read Archive (SRA) under the accession number SRP158342 and SRP158636, respectively.

## Results

### Determination of Bacterial and Archaeal Abundance

Real-time qPCR was performed to estimate the bacterial and archaeal 16S rRNA gene copy numbers that can be considered as an agent for bacterial and archaeal biomass (Table 2). Each mountain site supported both bacterial and archaeal phylotypes. The total number in CP14 (archaea plus bacteria) was 1.88 × 10^7^ 16S rRNA gene copies/g soil (wet weight), which was 1–2 orders of magnitude greater than the values for EH7 soils (5.93 × 10^6^ copies/g soil) and GU7 soils (4.68 × 10^5^ copies/g soil). The archaeal 16S rRNA gene copy numbers made up a relatively minor proportion (0.4–8.6%) of the total number of 16S rRNA gene copies in each soil sample. Hence, the microbial populations at the Union Glacier sites were all bacteria-dominated communities.

**TABLE 2:** Biodiversity indicies of bacterial communities and16S rRNA gene copies from the Union Glacier area.

**Sample**	**Reads**	**OTUs**	**Chao 1**	**Shannon**	**Pielou’s evenness**	**Good’s coverage**	**Bacteria (copy/g)**	**Archaea (copy/g)**
CP14	60,608	144	146.33	1.98	0.40	0.95	1.87 × 10^7^	1.86 × 10^5^
GU7	84,399	54	61.00	1.30	0.33	0.87	n.a.	n.a.
GU8	75,891	91	96.50	1.81	0.40	0.87	4.31 × 10^5^	3.73 × 10^4^
EH2	79,671	37	41.67	0.10	0.03	0.78	n.a.	n.a.
EH5	89,383	77	79.63	0.88	0.05	0.91	n.a.	n.a.
EH7	102,593	66	75.75	0.21	0.20	0.80	5.91 × 10^6^	2.27 × 10^4^

**n.a., no analysis.**

### Taxonomic Structure of Microbial Communities

A total of 492,545 high-quality reads were retained from all samples after filtering. In total, 212 OTUs were assigned with an average length of approximately 420 bp (Table 2). The number of OTUs changed from 37 to 144 across all samples, with the highest OTU numbers in CP14. There were strong differences in Shannon diversity and Pielou’s evenness indicies between samples. Furthermore, the bacterial diversity in Elephant Head sample was significantly lower than that in those from Rossman Cove and Charles Peak.

**FIGURE 2:**
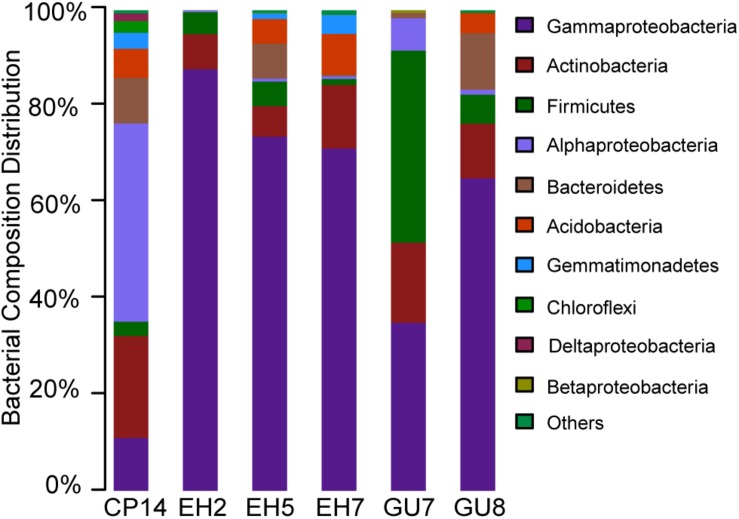
Bacterial composition distributions at the phylum level across all samples in the Union Glacier area. Sequences were assigned in the RDP reference database by using a 80% confidence cut-off.

The 212 OTUs could be grouped into 127 genera, representing 11 different phyla. Proteobacteria were predominant throughout the soil and rock samples (66.0%, on average), therein, the class Gammaproteobacteria dominated. Two other phyla, Firmicutes and Actinobacteria, were also distributed in all samples. Firmicutes showed a much higher abundance in subsurface soils relative to surface soils. The relative abundance of Actinobacteria, ranging from 1.2 to 39.9%, was the highest in GU7. The phylum Bacteroidetes was mainly distributed in surface soils of GU8 (11.7%) but its relative abundance varied greatly. Acidobacteria and Gemmatimonadetes were also found in high abundance across most samples (Figure 2).

To identify phylogenetic differences at a finer taxonomic level, bacterial abundance variations were analyzed at genus level (Supplementary Figure S1). Less than 7.9% of all observed genera were present in all samples and their abundances varied greatly (Supplementary Table S4). As most abundant genus, *Halomonas* was mainly present in the EH group (52.4%, average relative abundance), which may be related to physical and chemical properties (e.g., salinity) of the soil and rock from the Elephant Head region. At the Rossman Cove summit, the genera *Halomonas* and *Lysobacter* were abundant in surface soils, accounting for 19.0 and 11.5%, respectively. *Anoxybacillus*, belonging to Firmicutes, made up 32.1% of the total bacterial communities in the subsurface soils. As a widespread member of the Bacteroidetes, *Flavobacterium* was abundant in GU8.

Phylogenetic annotations of metagenomic reads were used to assess the overall taxonomic community structure. Bacteria clearly dominated the composition of the microbial communities. More than 91% of all taxonomically assigned metagenomic reads matched bacteria, with a few representatives of archaea (1%) and eukaryotes (8%). The low abundance of archaeal reads was in agreement with the qPCR result where 16S rRNA gene copies of archaeal cells were numerically two orders of magnitude lower than bacterial cells. Within bacterial taxa, Proteobacteria still constituted a major fraction (52.9%), followed by Actinobacteria (11.1%), Acidobacteria (9.7%), Bacteroidetes (9.1%) and Firmicutes (4.9%). The bacterial taxonomic composition, provided by the metagenomic determined functional genes, was in agreement with the 16S rRNA gene amplicon taxonomic profiling of EH7 (Figure 2 and Supplementary Figure S1). Most of the archaeal metagenomic reads matched Euryarchaeota members (80.5%). *Methanosarcina* was found to be the most abundant with 13.3% of the sequences assigned, followed by *Methanocella* and *Methanothrix* (<5%). Additionally, representatives of Crenarchaeota and Thaumarchaeota were also abundant (8.0 and 6.7%). Here, differences in bacterial abundance between amplicon and metagenomic taxonomic profiling were detected. These differences probably arose because an amplicon in complex DNA mixtures can lead to different biases and distort original 16S rRNA gene ratios (Vavourakis et al., 2016).

### Beta-Diversity and Network Analyses of Microbial Communities

**FIGURE 3:**
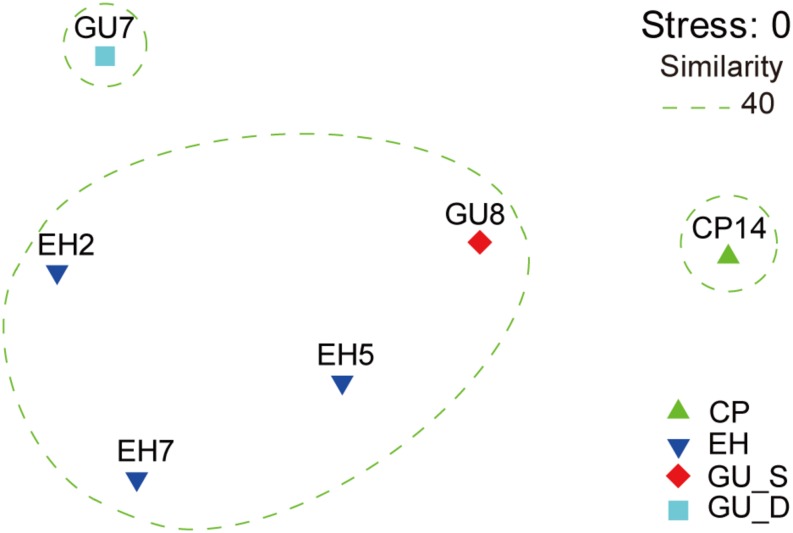
The non-metric multidimensional scaling (NMDS) of community similarities with the relative abundance of OTUs in the Union Glacier area. The ordination was built based on the rank order of bacterial Bray-Curtis similarity. All samples were divided into four groups: CP, EH, GU_S, and GU_D.

**FIGURE 4:**
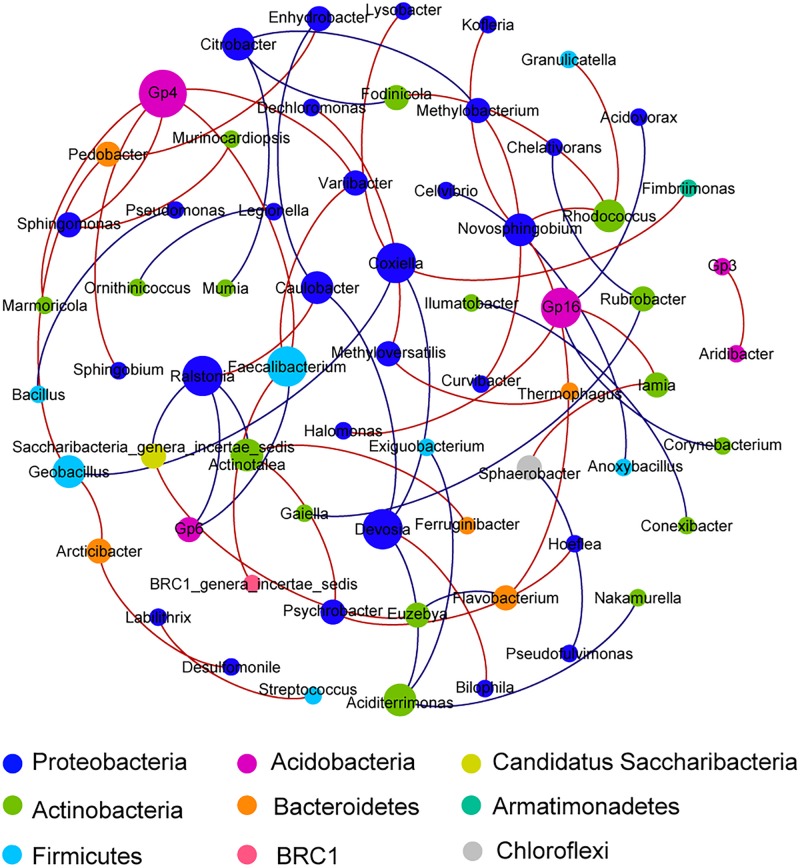
A network of co-occurrence pattern at genus level from all samples. Each line represents a significant correlation between two nodes; the red lines represent positive correlations, while the blue lines represent negative correlations. The size of each node is proportional to the number of connections. Correlations were identified by SparCC’s coefficient >0.65 or <−0.65 and *P* < 0.01, with each node representing a bacterial genus and the color representing the phylum that the genus is affiliated with.

The unconstrained non-metric MDS analysis was performed on the relative abundance of species in bacterial communities (Figure 3). The six samples divided into four groups, which showed significant community differences and there was a clear difference in community structures between groups (Supplementary Figure S2). At a 40% similarity level, four groups were clustered into three larger groups, which respectively were located in the three mountain sites. The microbial network, which was mainly comprised of two community groups, showed 65 nodes and 57 edges (Figure 4). Most of the positive correlations were detected between members of Proteobacteria, Actinobacteria and Firmicutes. The high abundances of Gp4, *Coxiella*, *Devosia*, *Faecalibacterium*, Gp16, and *Ralstonia* indicated a high incidence of inter- and intra-phylum correlations. Interestingly, chemoautotrophic *Methylobacterium* members exhibited interactions with other heterotrophic bacteria. In addition, Flavobacteriales communities had wider positive correlations with members from other phyla, likely illustrating some synergistic heterotrophic microorganisms potentially grew partly due to Flavobacteriia-derived organic products (Bunse and Pinhassi, 2017).

### Functional Structure of the Microbial Communities

The raw metagenomic reads were assembled into 167,519 contigs, which comprised 418,938 predicted genes. Based on the GhostKOALA analysis, 99.9% of metagenomic data were taxonomically assigned to Eukaryota and Prokaryote, while 45.0% could be assigned a KO number and used for subsequent functional analyses. Here, the marker genes of prokaryotes from identified KOs were focused on for a detailed study of carbon, nitrogen and sulfur cycling and stress responses (Supplementary Tables S1–S3). 285,522 (68.2%) of all predicted genes were assigned through an EggNOG database (Supplementary Figure S3). Among the known functional categories, the replication, recombination and repair (L) was the most abundant functional category, with 7.8% relative abundance. The second most abundant functional category was amino acid transport and metabolism (E) with a relative abundance of 7.0%, followed by cell wall/membrane/envelope biogenesis (M) (6.3%) and signal transduction mechanisms (T) (5.6%), respectively.

### Carbon Metabolism

**FIGURE 5:**
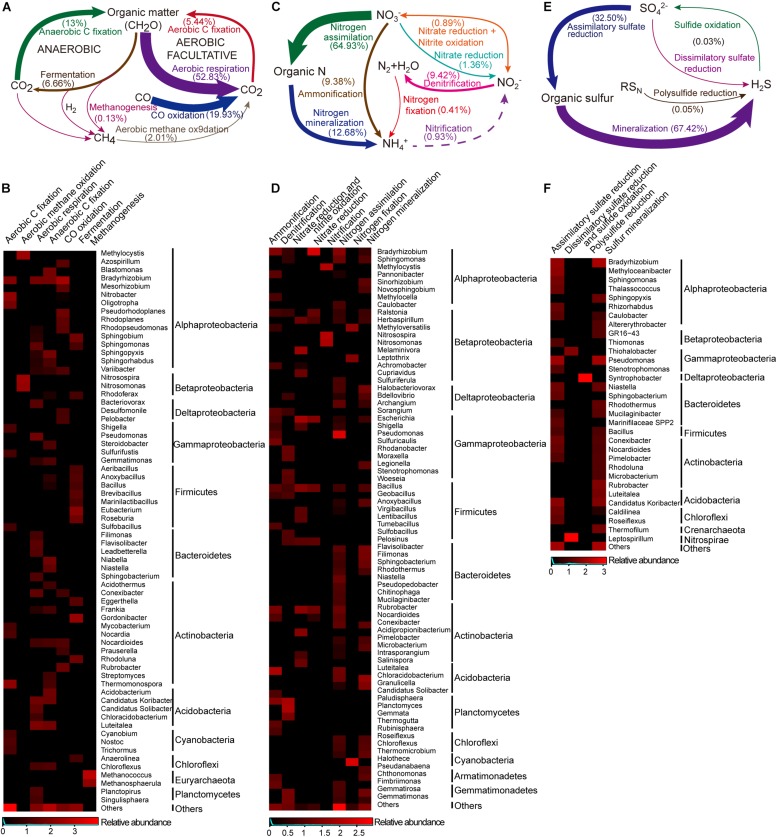
The carbon, nitrogen and sulfur cycles and their major taxa in the Union Glacier area. The genetic potential for each conversion step was estimated using a combination of normalized marker genes (Supplementary Table S1). Arrow size is proportional to the total potential flux (Supplementary Table S6). Heatmap presented the relative abundances of major microbes (only for those that contributed >1% of the marker genes mixture) potentially driving each conversion step. Color bars represent the row-scaled value, in which a blue curve illustrates the distribution density percentage. **(A)** Carbon cycle and **(B)** its major taxa. **(C)** Nitrogen cycle and **(D)** its major taxa. Dotted lines represent not detected marked genes but putative presence of the pathway. **(E)** Sulfur cycle and **(F)** its major taxa.

The main pathway for the carbon cycle was aerobic respiration, primarily associated with the heterotrophic genus *Luteitalea* (Acidobacteria) and the genus *Bradyrhizobium* (Alphaproteobacteria) (Figures 5A,B). In the aerobic carbon fixation, RuBisCO and Phosphoribulokinase (PRK) genes were found and affiliated to phototrophic and chemoautotrophic bacteria. Phototrophic Cyanobacteria communities [*Trichormus* and *Nostoc* (Nostocales) and *Cyanobium* (Synechococcales)] were found and these potentially used light energy to fix CO_2_ via the Calvin cycle. *Thermomonospora*, *Nitrobacter*, and *Bradyrhizobium* taxa comprised a predominant proportion of potential chemolithotrophic aerobic carbon fixers. By contrast, anaerobic carbon fixation was abundant with different types of pathway, including anoxygenic phototrophy by the Calvin cycle (Chromatiales), probably anaplerotic (Bacteroidales) and reductive citric acid cycle [sulfate-reducing bacteria (SRB)] (Lauro et al., 2011; Llorens-Marès et al., 2015). Another anoxygenic pathway, fermentation, was found to mineralize organic carbon into small molecules, and ultimately to CO_2_, this was mostly undertaken by the Actinobacteria and Firmicutes communities. However, these organic compounds can be broken into CO through incomplete biotic oxidation. Consistent with this, CO dehydrogenase genes were significantly present (20% of selected marker genes for carbon cycle). The phylogenetic affiliation of these marker genes was primarily to the genus *Bradyrhizobium*. In addition, both methanogenesis and methane oxidation marker genes were present at low abundances. Methanogenic archaea (e.g., *Methanosphaerula* and *Methanococcus*) were detected, while methanogenic bacteria were not found. The genes involved in methane oxidation were affiliated with the genera *Methylocystis*, *Nitrosospira*, and *Nitrosomonas*.

### Nitrogen Metabolism

**FIGURE 6:**
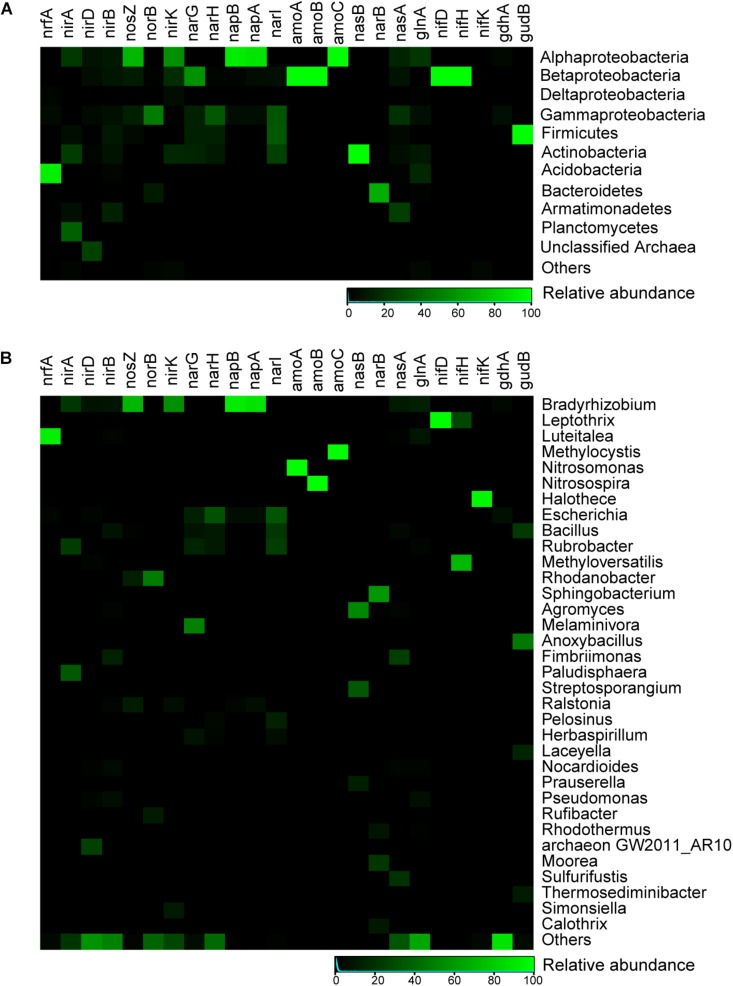
Heatmap showing relative abundances for main microbial taxa of selected key genes related to nitrogen metabolism in the Union Glacier area. **(A)** At the phylum level. **(B)** At the genus level. Color bars represent the row-scaled value, in which a blue curve illustrates the distribution density percentage.

For the nitrogen cycle, most of detected marker genes were related to nitrogen assimilation, followed by mineralization (Figure 5C). In the pathways related to exogenous ammonia input, high abundances of *GS* (glutamine synthetase) and *gdh* (glutamate dehydrogenase) were detected (Supplementary Figure S4). A complete set of denitrification genes [*nosZ* (nitrous oxide reductase), *norB* (nitric-oxide reductase) and *nirK* (nitrite reductase)] were found (9.4%) in the selected nitrogen functional reads. *Rhodanobacter* and *Bradyrhizobium* comprised a large proportion of potential denitrifiers (55.8%) (Figures 5D, 6). However, a potentially complete nitrification pathway was also found, in spite of lacking the *hao* (hydroxylamine dehydrogenase) gene. Aerobic ammonia oxidation made up a low percentage (0.9%) of overall nitrogen pathways. Potential ammonia oxidizers were assigned to *Nitrosomonas* and *Nitrosospira* (Nitrosomonadales, ammonia-oxidizing bacteria, AOB) and *Methylocysti* (Alphaproteobacteria). A low abundance (0.07% of total metagenomic reads) of ammonia-oxidizing archaea (AOA) was found, based on phylogenetic annotation of metagenomic functional reads. However, ammonia-oxidizing bacteria (Nitrobacter-, Nitrosospira-, Nitrosomonas-, and Nitrosococcus-like) and nitrite-oxidizing bacteria (Nitrospirae-like) metagenomic reads were detected at a 10 times higher abundance (1% of total reads). Unlike marker genes for aerobic ammonia oxidation, anammox marker genes coding for hydrazine synthase and dehydrogenase were not found. Additionally, with a low abundance (0.4% of the total selected nitrogen functional pathways), nitrogen fixation genes was associated with *Halothece* (Cyanobacteria), *Methyloversatilis*, and *Leptothrix* (Betaproteobacteria) and *Methanococcus* (Euryarchaeota) (Figures 5D,6).

### Sulfur Metabolism

Sulfur mineralization and assimilatory sulfate reduction marker genes were abundant (67.4 and 32.5%) among sulfur metabolic pathways, and 34.7 and 23.8% of marker genes in both pathways potentially originated from heterotrophic *Bradyrhizobium* and *Pseudomonas*, respectively (Figures 5E,F). The marker genes associated with dissimilatory sulfate reduction were found and attached to the genus *Leptospirillum* (Nitrospirae). Sulfide oxidation marker genes were assigned to *Thiohalobacter* (Gammaproteobacteria). A complete sulfur oxidation (Sox) system, by which thiosulfate was converted to sulfate, was also detected. Based on the metagenomic taxonomic profiling, a large diversity of potential heterotrophic sulfur oxidizers were identified and classified as green sulfur bacteria (GSB), within the order Chlorobiales, photosynthetic sulfur bacteria (PSB), within the family Chromatiaceae, and purple non-sulfur bacteria (PNSB), within the family Rhodobacteraceae (Schütte et al., 2016). Additionally, sulfur-reducing *Geobacter* was the most abundant of the sulfur reducers, followed by sulfur-reducing *Desulfuromonas*, sulfate-reducing *Desulfobulbus*, and sulfate-, sulfite-, and sulfur-reducing Desulfobacteraceae (Schütte et al., 2016). Interestingly, *Desulfomonile* (Syntrophobacterales), which not only acts as a heterotrophic sulfate reducer but also as a sulfide oxidizer was found (Llorens-Marès et al., 2015). Polysulfide reduction, which is driven by *Syntrophobacter* (Deltaproteobacteria) was rare.

### Adaption to Extreme Environment Stress

**FIGURE 7:**
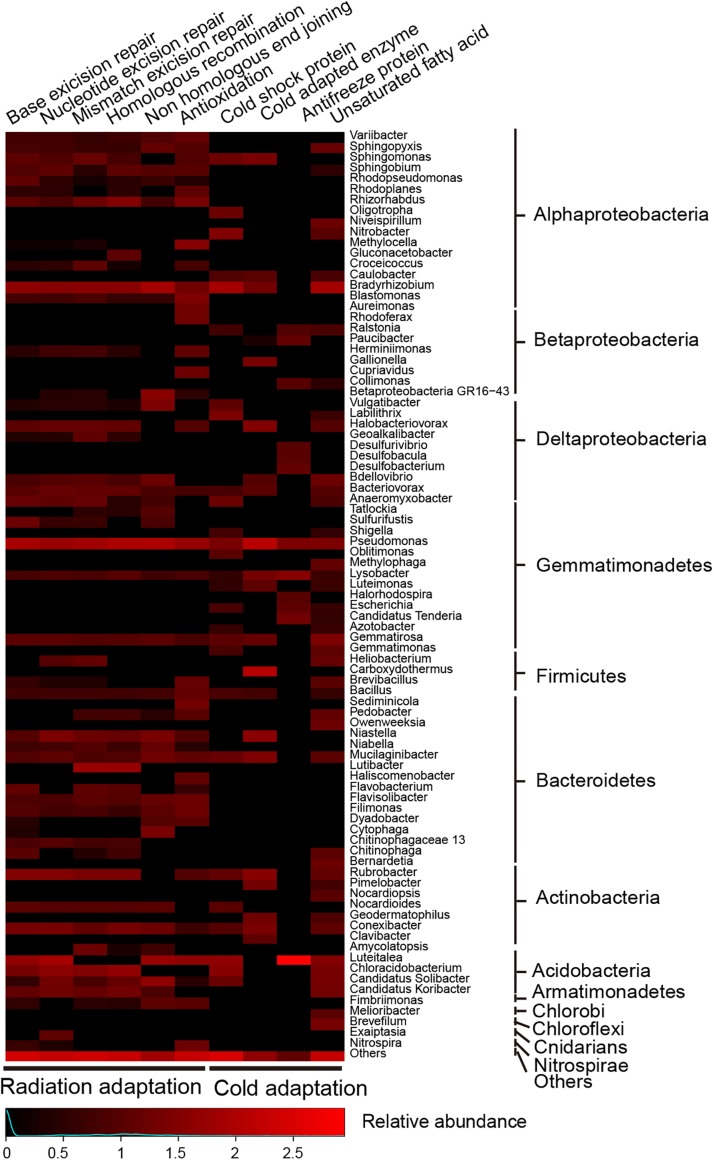
Heatmap showing relative abundances for main microbes (only for those that contributed >1% of the marker genes mixture) potentially driving DNA repair, antioxidation and cold adaption to an extremely low temperature in the Union Glacier area. Color bars represent the row-scaled value, in which a blue curve illustrates the distribution density percentage.

Different metabolic pathways which are related to adaptation to Antarctic extreme environments were observed in the metagenomic data (Figure 7). For adaptation to high solar radiation, the complete DNA repair pathway was detected in abundance (1.46% of total prokaryotic gene abundance). Different DNA repair forms, such as base excision repair, nucleotide excision repair, mismatch excision repair, homologous recombination and non-homologous end-joining, were all detected. Superoxide dismutase, catalase, and catalase-peroxidase genes have been found to play important roles in antioxidation. With regard to the adaptation to low temperature, cold-related proteins, cold-adapted enzymes and unsaturated fatty acid biosynthesis were observed in the metagenomic data. The genes related to adaptation to high radiation and low temperature were found in diverse range of microorganisms, indicating that the adaptation to extreme conditions is prevalent in Antarctic mountain ecosystems. Taxa related to acid and osmotic adaptations were also potentially identified (Supplementary Table S5).

## Discussion

Though Baeza et al. reported the microfungal diversity in the terrestrial habitats of the Union Glacier regions (Baeza et al., 2017). In this study, we further explored the microbial community structure and their potential functions in high altitude mountain soils of this region. A large variety of putative phototrophic and chemolithotrophic organisms, including sulfide-oxidizing and sulfate-reducing bacteria, fermenters, denitrifiers, methanogens, methane oxidizers, carbon and nitrogen fixers were found. The linkage between important biogeochemical metabolic pathways and their phylogenetic identities revealed how putative functional ecology was driven by these microorganisms.

### Low Abundance and Diversity in Microbial Communities

Antarctic soils typically contain low numbers of microbes (Boetius et al., 2015). Interestingly, cell numbers in soils sampled in this study were around 10^5^–10^7^ per gram. Compared with this, biomass in the Antarctic Dry Valleys was about 10^6^–10^8^ cells g^–1^ soil (Cowan, 2009; Stomeo et al., 2012). Other Antarctica terrestrial niches, such as hypolith, chasmolith, and endolith, have higher biomass (10^7^–10^9^ cells g^–1^) than soils we sampled (Pointing et al., 2009). Totally 212 OTUs were found in the samples examined herein, which suggested a relatively low biodiversity compared to microbial communities of temperate and tropical environments (Fierer and Jackson, 2006). The Union Glacier area is exposed to high levels of ultraviolet radiation and very low temperatures (Madigan, 2012; Boetius et al., 2015) and these are likely to be responsible for the lower biomass and biodiversity (Pearce et al., 2012).

Bacteria numerically dominated in this soil habitat, as reported in the other cryospheres (sea ice, supraglacial, and subglacial habitats) (Boetius et al., 2015). These bacterial communities harbored primarily Proteobacteria, along with Actinobacteria, Firmicutes, and Bacteroidetes. The similar observation was found in other extreme environments, e.g., Antarctic supraglacial habitats, high Alpine mountains, and Arctic soils (Boetius et al., 2015; Malard and Pearce, 2018). Inconsistently, in the McMurdo Dry Valleys, Acidobacteria, and Actinobacteria dominated soil bacterial communities, which may be related to soil characteristics (Pointing et al., 2009; Niederberger et al., 2019). Gammaproteobacteria constituted a major fraction of Proteobacteria in this study, which also were often found in Arctic and Antarctic sea ice (Boetius et al., 2015). However, high Alpine snow microbes were dominated by Alphaproteobacteria and Betaproteobacteria (Wunderlin et al., 2016; Azzoni et al., 2018). In this study, occurrence of Cyanobacteria and Chloroflexi members in soils indicated a true soil community may indeed exist in this barren region (Pointing et al., 2009). Flavobacteriales members were found to degrade high molecular weight organic matter into smaller molecules for the benefit of other heterotrophic microorganisms (Figure 4; Bunse and Pinhassi, 2017). These interactions between trophic microbes, resulting in the transformation of organic matter, could be likely to contribute to the formation of a complete ecosystem in this area.

### Carbon and Nitrogen Fixation by Autotrophic Microorganisms

In this study, Cyanobacteria, an important primary producer in different niches of cryosphere (Boetius et al., 2015), was found to participate in aerobic carbon fixation. Some Cyanobacteria members have the capacity to upregulate carbon concentrating mechanisms (CCM) to enhance the acquisition of inorganic carbon (CO_2_ or bicarbonate) (Price, 2011). Carboxysomes use the paradigm of metabolic channeling to enhance the local CO_2_ concentrations and increase the efficiency of RuBisCO (Lin et al., 2014). In this study, four genes related to carboxysome structure were identified. Additionally, a nitrifier, *Nitrobacter*, which uses energy from the oxidation of nitrite to fix CO_2_ via the Calvin cycle (Fiencke et al., 2005), was observed in this study. Another chemolitoautotrophic nitrifier, *Nitrolancea* (Chloroflexi), which participates in aerobic carbon fixation and interacts with other bacteria, was identified in the network analysis (Figure 4). As reported previously, these photoautotrophs and chemoautotrophs functioned during sunlight or dark conditions and are the basis for complex food webs in extreme environments where plants barely exist (Boetius et al., 2015; Malard and Pearce, 2018).

The reverse tricarboxylic acid (rTCA) cycle and anaerobic C1-pathway have been reported to be the main pathways for anaerobic carbon fixation in the hydrothermal chimney (He et al., 2015). In this study, a high percentage of anaerobic carbon fixation compared to aerobic carbon fixation was observed. The most likely organisms responsible for chemotrophic CO_2_ fixation were Chromatiales, sulfate-reducing bacteria (SRB) and Chlorobiales (GSB), which were also found in Antarctic Ace Lake (Cavicchioli, 2015). Desulfobacterales, which were found here, undertake formate assimilation and CO_2_ fixation through anaerobic C1-pathway (Fuchs, 2011). Sulfate-reducing bacteria (e.g., *Pelobacter* and *Desulfomonile*) were detected herein, which have the potential to fix carbon and degrade a wide variety of carbon compounds (Llorens-Marès et al., 2015). Therefore, in combination with sulfur and nitrogen metabolism, the data presented here suggests that inorganic carbon fixation provided a carbon source and energy for the survival of these polar microbial populations.

A key process in nitrogen cycling is nitrogen fixation, which were found to be primarily driven by diazotrophic cyanobacterial taxa in this study. Cyanobacteria from Antarctic hypolithic subsurface soils have also been found to be the dominant contributors to nitrogen fixation (Cowan et al., 2011). Allen et al. (2009) further reported that methanogens, such as *M. burtonii*, could benefit from nitrogen fixation. Consistently, nif genes of the methanogen, *Methanococcus*, were detected in the metagenomic data reported herein. Hence, evidence for symbiotic relationships between diazotrophic archaea and methanogens was tentatively found, as reported previously (Lauro et al., 2011). *Methyloversatilis* (Nitrosomonadales) was detected among diazotrophic taxa herein, although it commonly acted as a methylotroph in the sediments (Kumaresan et al., 2018). It was reported that Methylotrophs can function in methane oxidation and carbon fixation through the C1-pathway (Kumaresan et al., 2018). This result suggests that the methylotroph *Methyloversatilis* could provide nitrogen and carbon for other microbes, this was demonstrated here in the interaction network analyses (Figure 4). *Bradyrhizobium*, from which a large number of denitrification genes were found in this study, has been identified as a chemolithoautotroph that can fix atmospheric nitrogen to form ammonia or ammonium (Hennecke, 1990).

### Other Metabolic Processes for Carbon, Nitrogen, and Sulfur

The incomplete biological oxidation of organic carbon compounds can lead to CO production. Several studies have found that the use of CO as an alternative energy and carbon source is widespread among heterotrophs (Cunliffe, 2010). For example, the heterotrophic species, *Halobacteria*, benefits from metabolizing CO in surface waters (Vavourakis et al., 2016). In this study, CO oxidation accounted for a large proportion (19.9%) in selected carbon pathways (Figure 5A and Supplementary Table S6). Interestingly, 2.9% of CO dehydrogenase genes were related to Sulfate-reducing bacteria *Pelobacter* and *Desulfomonile*. This may be because CO oxidation was probably coupled to sulfate reduction or CO_2_ reduction to produce energy-yielding substrates such as sulfide, H_2_ and acetate (Llorens-Marès et al., 2015). It was also found that only a low proportion of genes (0.1%) were associated with methanogenic archaea. Aerobic methane oxidation was also rare. Surprisingly, typical ammonia oxidizers, such as *Nitrosospira* and *Nitrosomonas*, were found to participate in methane oxidation. Fiencke et al. (2005) have suggested that *Nitrosomonas* can use energy gained from the oxidation of ammonia to fix CO_2_. This illustrates that CO_2_ fixation, methane oxidation and ammonia oxidation may function together in polar high altitude soils.

In this study most of the genetic nitrogen pathways were associated with nitrogen assimilation and remineralization. Glutamine synthetase, glutamate synthase and glutamate dehydrogenase were detected herein and have been recorded through the metaproteome in Antarctic Ace Lake, which implied that active nitrogen assimilation and remineralization happened (Lauro et al., 2011). The rate of nitrification was restricted by the first step, i.e., oxidation of ammonia to nitrite, which was driven by AOA and AOB. A low abundance of ammonia oxidizers (both AOA and AOB) was found in the metagenomic pool. Additionally, a significantly higher abundance of AOB with respect to AOA was found. Unlike known ammonia-oxidizing bacteria, several ammonia-oxidizing archaea were also reported that could potentially sustain high specific oxidation rates under extreme oligotrophic ammonium limitation (Martens-Habbena et al., 2009). The high affinity of AOA for oligotrophic ammonium could compensate for their low abundance and further illustrates that AOA could potentially compete with heterotrophic bacteria in this extreme environment.

The main turnover of sulfur compounds occurred within the sulfur mineralization process, suggesting that numerous heterotrophic microorganisms potentially degraded organic sulfur compounds into small inorganic molecules. *Thiohalobacter* was detected to probably oxidize sulfide in the polar mountain soils examined herein, which agreed with the research that the *Thiohalobacter* group was a moderately halophilic, sulfur-oxidizing community and some species of this could degrade thiocyanate aerobically (Sorokin et al., 2010). Genes for dissimilatory sulfate reduction were present in the genus *Leptospirillum*. Previous studies have shown that the *Leptospirillum* group is typical acidophilic and iron-oxidizing communities, which play a key role in bioleaching and biooxidation (Smith and Johnson, 2018). Also, *Leptospirillum* is strictly chemolithoautotrophic and able to fix carbon using ferrous iron as its electron donor and oxygen as the electron acceptor (Mi et al., 2011). Some phototrophic microorganisms, such as the GSB and PSB groups, which are potential sulfur oxidizers, were found here. It has been suggested that PSB can oxidize a variety of reduced sulfur species (e.g., sulfide, thiosulfate, and elemental sulfur) (Schütte et al., 2016). Similarly, GSB typically oxidize sulfide and thiosulfate but has lost essential genes for elemental sulfur oxidation (Gregersen et al., 2011). Both PSB and GSB carry out anoxygenic photosynthesis using reduced sulfur, implying that they were able to coordinate the sulfur and carbon cycles (Llorens-Marès et al., 2015).

### Extreme Environments Adaptation

Some microorganisms have adapted to living in environments exposed to high levels of solar irradiation by a combination of mechanisms, e.g., efficient DNA repair, high resistance to oxygen-free radicals through production of superoxide dismutase and other special survival modes (Martín-Cerezo et al., 2015). In this study, several complete metabolic pathways of DNA repair and antioxidation genes were detected in a range of microorganisms. These phyla were common and occurred generally in different niches (Chan et al., 2013). Extreme low temperatures, such as experienced in the Union Glacier area, is one of the most challenging stresses for life on Earth (Garcia-Lopez and Cid, 2017). However, microorganisms have developed complex metabolic strategies for survival at low temperatures (Madigan, 2012; Boetius et al., 2015). Genes for cold adaptions were found in this study. In addition, microorganisms from the Union Glacier area were expected to synthesize glycine/betaine/proline compounds, osmoprotectants and various osmotic proteins to improve their adaption at high salinity. These taxa mainly included Proteobacteria and Actinobacteria, which was also found in Antarctic rocky substrates (Chan et al., 2013). Sometimes, unique microorganisms (e.g., xerophiles and acidophiles) can tolerate desiccation and extremely acidic conditions (Imshenetsky et al., 1973; Cid et al., 2010). Here, it is clearly shown that the microbial communities had abundant and diverse stress response pathways. The similar observations have been detected for osmotic, radiation, desiccation and cold stress from soils, hypoliths, chasmoendoliths and cryptoendolith in Dry Valley niches (Chan et al., 2013). These genes and pathways would have equipped the communities with a diverse potential “arsenal” of responses to environmental extremes allowing them to survive in this very inhospitable terrain.

## Conclusion

The metagenomic analysis demonstrated the relationships between microbial communities and biogeochemical cycling in this Antarctic mountain soils. A low biomass and diversity was observed, probably due to the severe local environment. Although archaea were detected, bacteria dominated. A diverse range of producers were found and the ecosystem was reconstructed based on analyses of carbon, nitrogen and sulfur metabolic pathways. Carbon fixation taxa were comprised of photoautotrophs (Cyanobacteria) and chemoautotrophs (mainly Alphaproteobacterial clades: *Bradyrhizobium*, *Sphingopyxis*, and *Nitrobacter*). The main nitrogen fixation taxa were *Halothece* (Cyanobacteria), *Methyloversatilis*, and *Leptothrix* (Betaproteobacteria). These putative producers would have been able to provide organic carbon and nitrogen and interact with other heterotrophic microbes. In addition, a number of complete metabolic pathways and genes associated with high radiation, low temperature and other stress adaptations were detected. These indicate that the microbial communities had adapted to this harsh environment and could survive. Next, together with *in situ* geochemical parameter measurements, more efforts will be spent on metatranscriptome and metaproteome analyses, which could further confirm and estimate metabolic potentials of the microbial community at an active level.

## Data Availability Statement

The datasets generated for this study can be found in the NCBI Sequence Read Archive, SRP158342 and SRP158636.

## Author Contributions

YL, Q-QC, and Y-RD performed the laboratory work. GE and JB collected and processed the environmental samples to obtain the genetic material and took the landscape photographies shown in Figure 1. YL wrote the manuscript. Q-LQ helped in data analysis. Q-LQ, X-LC, MW, and AM helped to revise the manuscript. Y-ZZ and JB designed the research.

## Conflict of Interest

The authors declare that the research was conducted in the absence of any commercial or financial relationships that could be construed as a potential conflict of interest.

## Footnotes

1 https://github.com/najoshi/sickle
