# Supplementary material for: Reconstruction of the Functional Ecosystem in the High Light, Low Temperature Union Glacier Region, Antarctica
Source: Front Microbiol. 2019 Oct 18;10:2408. doi: 10.3389/fmicb.2019.02408 (PMC6813960; doi:10.3389/fmicb.2019.02408)
Supplement: Supplementary file 1 [file Data_Sheet_1.docx]

**Supplementary Material**

**Reconstruction of the functional ecosystem in the high light, low temperature Union Glacier region, Antarctica**

Yi Li^1^, Qian-Qian Cha^1^, Yan-Ru Dang^1^, Xiu-Lan Chen^1,3^, Min Wang^2^, Andrew McMinn^2,4^, Giannina Espina^5^, Yu-Zhong Zhang^1,2,3^, Jenny M. Blamey^5,6^*, Qi-Long Qin^1^*

^1^State Key Laboratory of Microbial Technology, Marine Biotechnology Research Center, Shandong University, Qingdao, China

^2^College of Marine Life Sciences, Institute for Advanced Ocean Study, Ocean University of China

^3^Laboratory for Marine Biology and Biotechnology, Qingdao National Laboratory for Marine Science and Technology, Qingdao, China

^4^Institute for Marine and Antarctic Studies, University of Tasmania, Hobart, Tasmania, Australia

^5^Fundación Científica y Cultural Biociencia, José Domingo Cañas, 2280, Santiago, Chile.

^6^Faculty of Chemistry and Biology, Universidad de Santiago de Chile, Santiago, Chile

* Corresponding author:

Qi-Long Qin, qinqilong@sdu.edu.cn; Jenny M. Blamey: jblamey@bioscience.cl

**Supplementary Figures and Tables**

**Supplementary Figures**

**
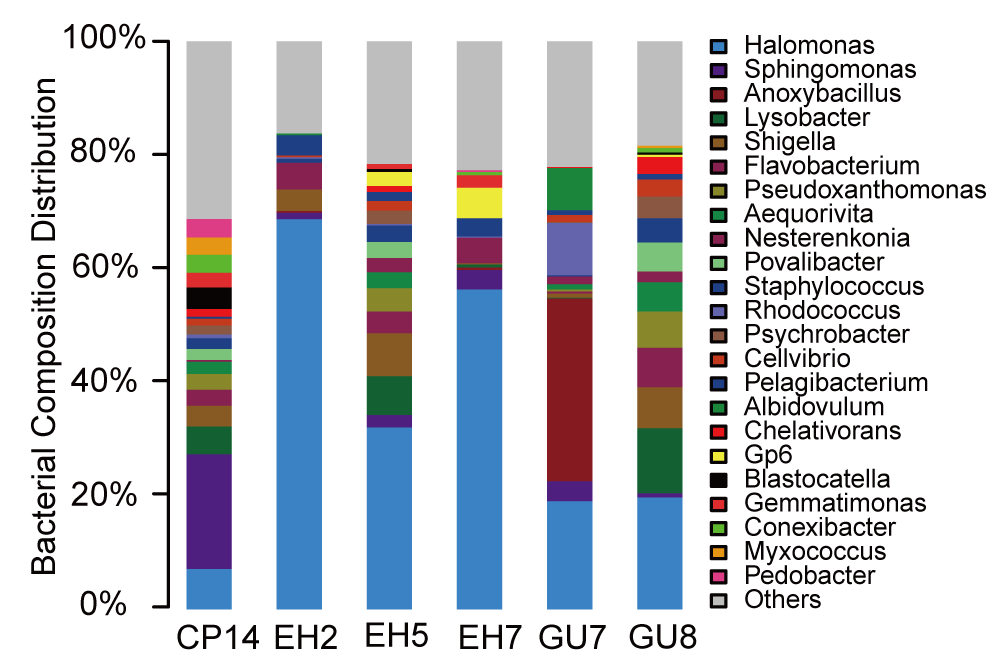
**

**Figure S1. Bacterial composition distributions at the genus level across all samples in the Union Glacier area.** Sequences were assigned in the RDP reference database by using a 80% confidence cut-off.


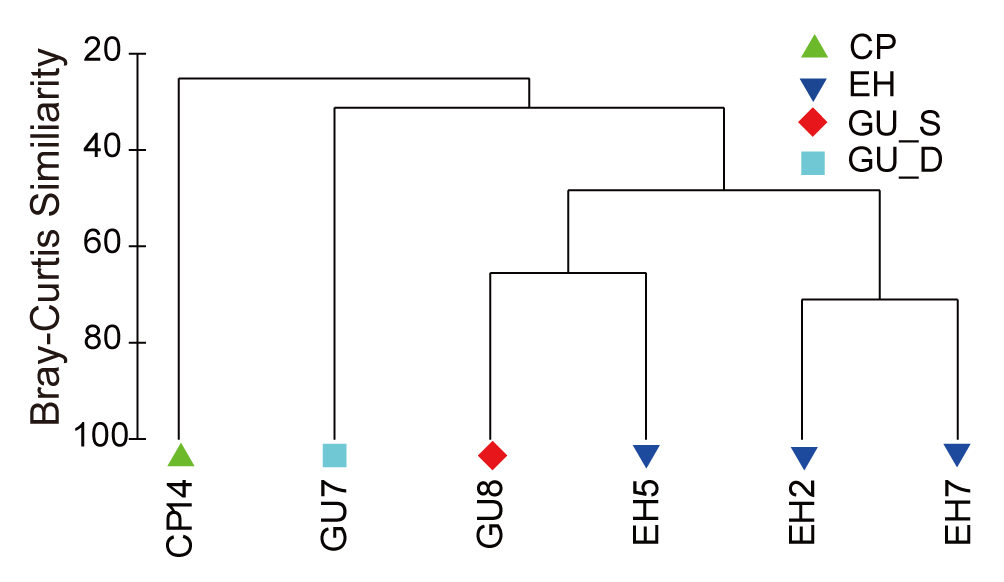


**Figure S2. The cluster dendrogram of community similarities among samples with the relative abundance of OTUs in the Union Glacier area.** The ordination was built based on the rank order of bacterial Bray-Curtis similarity. All samples were divided into four groups: CP, EH, GU_S and GU_D.


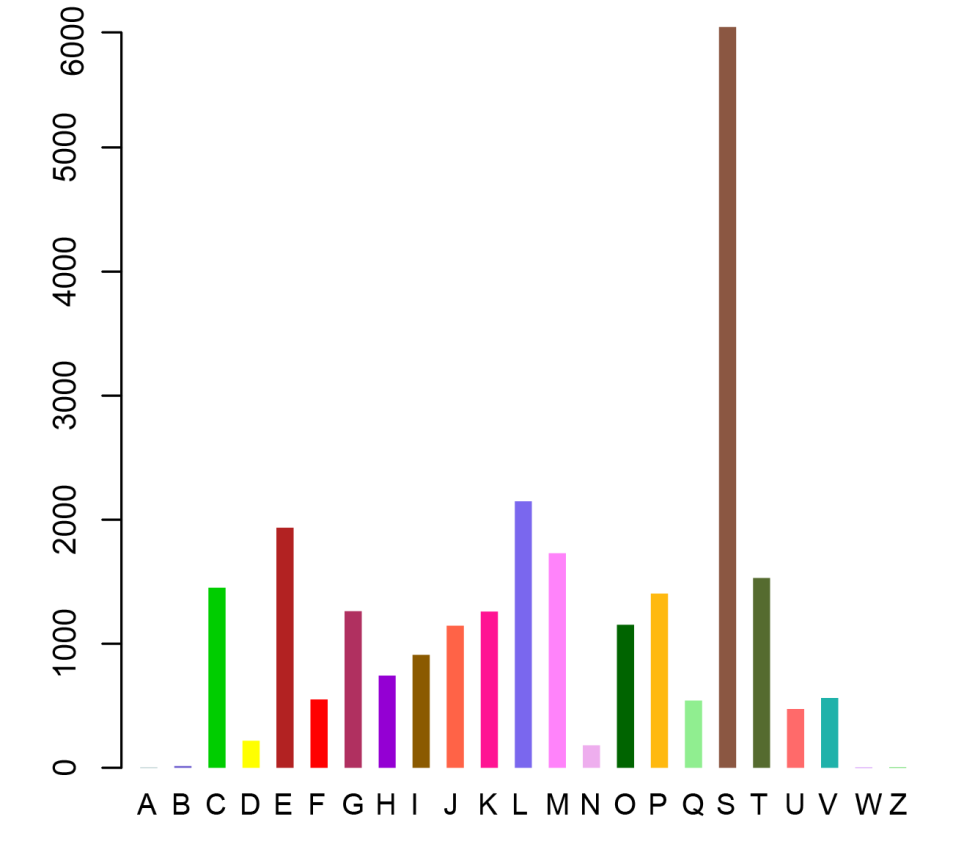


**Figure S3. COG functional categories of the predicted open reading frames (ORFs) from the metagenomic data through diamond searches by eggNOG-mapper in the Union Glacier area.** ORFs are depicted in different colors according to COG categories: RNA processing and modification (A); Chromatin structure and dynamics (B); Energy production and conversion (C); Cell cycle control, cell division and chromosome partitioning (D); Amino acid transport and metabolism (E); Nucleotide transport and metabolism (F); Carbohydrate transport and metabolism (G); Coenzyme transport and metabolism (H); Lipid transport and metabolism (I); Translation, ribosomal structure and biogenesis (J); Transcription (K); Replication, recombination and repair (L); Cell wall/membrane/envelope biogenesis (M); Cell motility (N); Posttranslational modification, protein turnover and chaperones (O); Inorganic ion transport and metabolism (P); Secondary metabolites biosynthesis, transport and catabolism (Q); Function unknown (S); Signal transduction mechanisms (T); Intracellular trafficking, secretion, and vesicular transport (U); defense mechanisms (V); Extracellular structures (W); Cytoskeleton (Z).


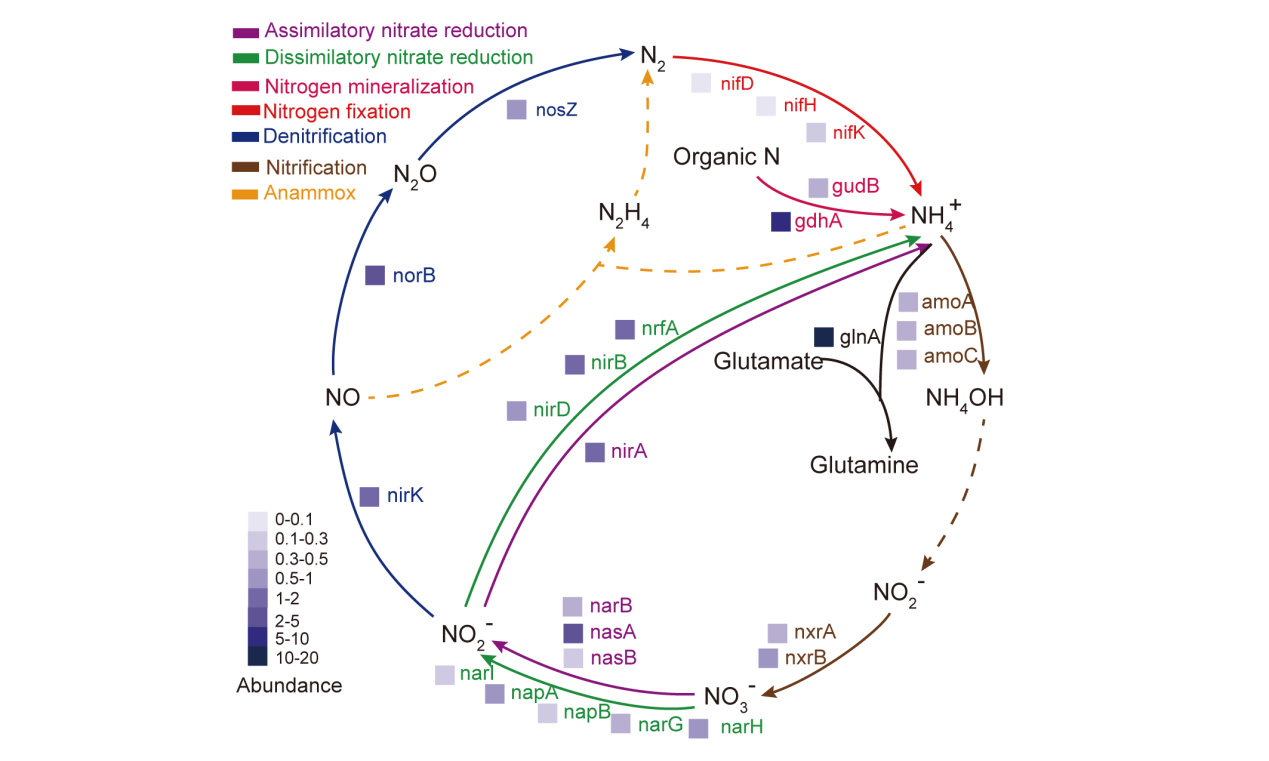
**Figure S4. The genetic potential for each step of the nitrogen metabolism in the Union Glacier area.** The potential for each nitrogen metabolism step was identified using relative abundance of marker genes. The color shade of each marker gene is proportional to its relative abundance. Dotted lines represent not detected marked genes but putative presence of the pathway.

**Supplementary Tables**

**Table S1. The marker genes for each conversion step of carbon, nitrogen and sulfur cycles in the Union Glacier area.**

|  | **Step** | **KEGG** | **Marker gene** |
| --- | --- | --- | --- |
| **Carbon** | **Aerobic C fixation***(Calvin cycle) K00855+(K01602+K01601)/2 | K00855 | Phosphoribulokinase (prkB) |
|  |  | K01601 | RuBisCO large chain (rbcL) |
|  |  | K01602 | RuBisCO small chain (rbcS) |
|  | **Aerobic methane oxidation**  (K10944+K10945+K10946)/3 | K10944 | methane monooxygenase subunit A (pmoA) |
|  |  | K10945 | methane monooxygenase subunit B (pmoB) |
|  |  | K10946 | methane monooxygenase subunit C (pmoC) |
|  | **Aerobic respiration** (K02256+K02262)/2+(K02274+  K02276)/2 | K02256 | cytochrome c oxidase subunit I (coxI) |
|  |  | K02262 | cytochrome c oxidase subunit III (coxIII) |
|  |  | K02274 | cytochrome c oxidase subunit I (coxA) |
|  |  | K02276 | cytochrome c oxidase subunit III (coxC) |
|  | **Anaerobic C fixation** (Arnon: K00174, K00175, K00244, K01648) (K00174+K00175+K00244+ K01648)/4 | K00174 | 2-oxoglutarate:ferredoxin oxidoreductase subunit alpha (korA/oorA/oforA) |
|  |  | K00175 | 2-oxoglutarate:ferredoxin oxidoreductase subunit beta (korB/ooBA/oforB) |
|  |  | K00244 | fumarate reductase flavoprotein subunit (frdA) |
|  |  | K01648 | ATP citrate lyase (ACLY) |
|  | **CO oxidation** (K03518+K03519+K03520)/3 | K03518 | CO dehydrogenase small subunit (coxS) |
|  |  | K03519 | carbon-monoxide dehydrogenase medium subunit (cutM/coxM) |
|  |  | K03520 | carbon-monoxide dehydrogenase large subunit (cutL/coxL) |
|  | **Fermentation** | K00016 | L-lactate dehydrogenase (LDH) |
|  | **Methanogenesis** | K00400 | coenzyme M methyl reductase beta subunit (mcrB) |
| **Nitrogen** | **Ammonification**  K03385+K00366+(K00362+K00363)/2 | K03385 | formate-dependent nitrite reductase periplasmic cytochrome c552 (nrfA) |
|  |  | K00362 | nitrite reductase (NADH) large subunit (nirB) |
|  |  | K00363 | nitrite reductase (NADH) small subunit (nirD) |
|  |  | K00366 | ferredoxin-nitrite reductase (nirA) |
|  | **Denitrification**  K00368+K04561+K00376 | K00376 | nitrous oxide reductase (nosZ) |
|  |  | K04561 | nitric-oxide reductase (norB) |
|  |  | K00368 | nitrite reductase (NO-forming) (nirK) |
|  | **Nitrate reduction + Nitrite oxidation** (K00370+K00371)/2 | K00370 | nitrate reductase alpha & nitrite oxidoreductase (narG/nxrA) |
|  |  | K00371 | nitrate reductase beta & nitrite oxidoreductase (narH/nxrB) |
|  | **Nitrate reduction**  (K02567+K02568)/2 + K00374 | K02567 | periplasmic nitrate reductase (napA) |
|  |  | K02568 | cytochrome c-type protein (napB) |
|  |  | K00374 | nitrate reductase gamma subunit (narI) |
|  | **Nitrification** (K10944+K10945+K10946)/3 | K10944 | ammonia monooxygenase subunit A (amoA) |
|  |  | K10945 | ammonia monooxygenase subunit B (amoB) |
|  |  | K10946 | ammonia monooxygenase subunit C (amoC) |
|  | **Nitrogen assimilation** (K00360+K00372)/2+K00367+K01915+ K00265+K00284 | K00265 | glutamate synthase (NADPH/NADH) large chain (gltB) |
|  |  | K00284 | glutamate synthase (ferredoxin-dependent) (gltS) |
|  |  | K00360 | assimilatory nitrate reductase electron transfer subunit (nasB) |
|  |  | K00367 | ferredoxin-nitrate reductase (narB) |
|  |  | K00372 | assimilatory nitrate reductase catalytic subunit (nasA) |
|  |  | K01915 | glutamine synthetase (glnA) |
|  | **Nitrogen Fixation** (K02586+K02588+K02591)/3 | K02586 | nitrogenase molybdenum-iron protein alpha chain (nifD) |
|  |  | K02588 | nitrogenase iron protein (nifH) |
|  |  | K02591 | nitrogenase molybdenum-iron protein beta chain (nifK) |
|  | **Nitrogen Mineralization** K00260+K00261+K00262 | K00260 | glutamate dehydrogenase (gudB) |
|  |  | K00261 | glutamate dehydrogenase (NAD(P)+) (gdhA) |
|  |  | K00262 | glutamate dehydrogenase (NADP+)(gdhA) |
| **Sulfur** | **Assimilatory sulfate reduction** (K00860+K00956+K00957+K00955)/4  +K00958 | K00860 | adenylylsulfate kinase (cysC) |
|  |  | K00955 | bifunctional enzyme CysN/CysC (cysNC) |
|  |  | K00956 | sulfate adenylyltransferase subunit 1 (cysN) |
|  |  | K00957 | sulfate adenylyltransferase subunit 2 (cysD) |
|  |  | K00958 | sulfate adenylyltransferase (sat) |
|  | **Dissimilatory sulfate reduction and sulfide oxidation****(K00394+K11180)/2 | K00394 | adenylylsulfate reductase subunit A (aprA) |
|  |  | K11180 | sulfite reductase (dsrA) |
|  | **Sulfur Mineralization** K00456+K01011+(K00380+K00381)/2+  K00390+ K00392 | K00456 | cysteine dioxygenase |
|  |  | K01011 | 3-mercaptopyruvate sulfurtransferase |
|  |  | K00380 | sulfite reductase (NADPH) flavoprotein alpha-component (cysJ) |
|  |  | K00381 | sulfite reductase (NADPH) hemoprotein beta-component (cysI) |
|  |  | K00390 | phosphoadenosine phosphosulfate reductase (cysH) |
|  |  | K00392 | sulfite reductase (ferredoxin) (sir) |
|  | **Polysulfide reduction** | K08352 | polysulfide reductase chain A (psrA) |

*: Chromatiales: anoxygenic phototrophy through the Calvin cycle.

**: As marker genes K00394, K00395, K11180 can operate in both an oxidative and a reductive way. They were assigned to the sulfate reduction or sulfide oxidation step if they had a best match within KEGG to an ortholog from a sulfate-reducing or sulfur-oxidizing clade.

**Table S2.** **The marker genes for each conversion step of DNA repair and antioxidation in the Union Glacier area.**

|  | **step** | **KEGG** | **Marker gene** |
| --- | --- | --- | --- |
| **Base excision repair** | **Lesion recognition and removal**  K03648+K03649+K21929+K01247+ K13529+K03652+K03575+K01246+ K10563 | K03648 | uracil-DNA glycosylase (UNG/UDG) |
|  |  | K03649 | double-stranded uracil-DNA glycosylase (mug) |
|  |  | K21929 | uracil-DNA glycosylase (udg) |
|  |  | K01247 | DNA-3-methyladenine glycosylase II (alkA) |
|  |  | K13529 | DNA-3-methyladenine glycosylase II (ada-alkA) |
|  |  | K03652 | DNA-3-methyladenine glycosylase (MPG) |
|  |  | K03575 | A/G-specific adenine glycosylase (mutY) |
|  |  | K01246 | DNA-3-methyladenine glycosylase I (tag) |
|  |  | K10563 | formamidopyrimidine-DNA glycosylase (mutM/fpg) |
|  | **Strand scission**  K05522+K10773+K01142+K01151+ K05982+ K07462 | K05522 | endonuclease VIII (nei) |
|  |  | K10773 | endonuclease III (NTH) |
|  |  | K01142 | exodeoxyribonuclease III (xthA) |
|  |  | K01151 | deoxyribonuclease IV (nfo) |
|  |  | K05982 | deoxyribonuclease V (nfi) |
|  |  | K07462 | single-stranded-DNA-specific exonuclease (recJ) |
|  | **Gap filling and strand displacement** | K02335 | DNA polymerase I (polA) |
|  | **Ligation** | K01972 | DNA ligase (NAD+) (ligA/ligB) |
| **Nucleotide excision repair** | **Damage recognition and incision**  K03723+ K03701+ K03702+ K03703 | K03723 | transcription-repair coupling factor (superfamily II helicase) (mfd) |
|  |  | K03701 | excinuclease ABC subunit A (uvrA) |
|  |  | K03702 | excinuclease ABC subunit B (uvrB) |
|  |  | K03703 | excinuclease ABC subunit C (uvrC) |
|  | **Excision and DNA synthesis**  K03657+K02335 | K03657 | DNA helicase II (uvrD/pcrA) |
|  |  | K02335 | DNA polymerase I (polA) |
|  | **Ligation** | K01972 | DNA ligase (NAD+) (ligA/ligB) |
| **Mismatch excision repair** | **Mismatch recognition and incision**  **(**K03555+K07456)/2+K03572+ K03573 | K03555 | DNA mismatch repair protein MutS (mutS) |
|  |  | K07456 | DNA mismatch repair protein MutS2 (mutS2) |
|  |  | K03572 | DNA mismatch repair protein MutL (mutL) |
|  |  | K03573 | DNA mismatch repair protein MutH (mutH) |
|  | **Excision**  K03657+K01141+K07462+  K10857+(K03601+K03602)/2 | K03657 | DNA helicase II (uvrD/pcrA) |
|  |  | K01141 | exodeoxyribonuclease I (sbcB/exoI) |
|  |  | K07462 | single-stranded-DNA-specific exonuclease (recJ) |
|  |  | K10857 | exodeoxyribonuclease X (exoX) |
|  |  | K03601 | exodeoxyribonuclease VII large subunit (xseA) |
|  |  | K03602 | exodeoxyribonuclease VII small subunit (xseB) |
|  | **DNA re-synthesis**  K03111+(K02337+K03763+K02338+ K02339+K02340+K02341+ K02342+ K02343+ K02344+ K02345)/10 | K03111 | single-strand DNA-binding protein (ssb) |
|  |  | K02337 | DNA polymerase III subunit alpha (dnaE) |
|  |  | K03763 | DNA polymerase III subunit alpha (polC) |
|  |  | K02338 | DNA polymerase III subunit beta (dnaN) |
|  |  | K02339 | DNA polymerase III subunit chi (holC) |
|  |  | K02340 | DNA polymerase III subunit delta (holA) |
|  |  | K02341 | DNA polymerase III subunit delta' (holB) |
|  |  | K02342 | DNA polymerase III subunit epsilon (dnaQ) |
|  |  | K02343 | DNA polymerase III subunit gamma/tau (dnaX) |
|  |  | K02344 | DNA polymerase III subunit psi (hold) |
|  |  | K02345 | DNA polymerase III subunit theta (holE) |
|  | **Ligation**  K01972+ K06223 | K01972 | DNA ligase (NAD+) (ligA/ligB) |
|  |  | K06223 | DNA adenine methylase (dam) |
| **Homologous recombination** | **Filament formation**  K03582+K03583+K03581+K07462+ K03111+K03553+K03629+K03584+ K06187 | K03582 | exodeoxyribonuclease V beta subunit (recB) |
|  |  | K03583 | exodeoxyribonuclease V gamma subunit (recC) |
|  |  | K03581 | exodeoxyribonuclease V alpha subunit (recD) |
|  |  | K07462 | single-stranded-DNA-specific exonuclease (recJ) |
|  |  | K03111 | single-strand DNA-binding protein (ssb) |
|  |  | K03553 | recombination protein RecA (recA) |
|  |  | K03629 | DNA replication and repair protein RecF (recF) |
|  |  | K03584 | DNA repair protein RecO (recO) |
|  |  | K06187 | recombination protein RecR (recR) |
|  | **Strand invasion and DNA synthesis**  K02335+(K02337+K03763+K02338+ K02339+K02340+K02341+ K02342+ K02343+ K02344+ K02345)/10 | K02335 | DNA polymerase I (polA) |
|  |  | K03111 | single-strand DNA-binding protein (ssb) |
|  |  | K02337 | DNA polymerase III subunit alpha (dnaE) |
|  |  | K03763 | DNA polymerase III subunit alpha (polC) |
|  |  | K02338 | DNA polymerase III subunit beta (dnaN) |
|  |  | K02339 | DNA polymerase III subunit chi (holC) |
|  |  | K02340 | DNA polymerase III subunit delta (holA) |
|  |  | K02341 | DNA polymerase III subunit delta' (holB) |
|  |  | K02342 | DNA polymerase III subunit epsilon (dnaQ) |
|  |  | K02343 | DNA polymerase III subunit gamma/tau (dnaX) |
|  |  | K02344 | DNA polymerase III subunit psi (hold) |
|  |  | K02345 | DNA polymerase III subunit theta (holE) |
|  | **Branch migration and resolution of Holliday junction**  K03550+K03551+K01159+K03655 | K03550 | holliday junction DNA helicase RuvA (ruvA) |
|  |  | K03551 | holliday junction DNA helicase RuvB (ruvB) |
|  |  | K01159 | crossover junction endodeoxyribonuclease RuvC (ruvC) |
|  |  | K03655 | ATP-dependent DNA helicase RecG (recG) |
|  | **Replication restart**  K04066+ K02686+K04067+K02317 | K04066 | primosomal protein N' (replication factor Y) (superfamily II helicase) (priA) |
|  |  | K02686 | primosomal replication protein N (priB) |
|  |  | K04067 | primosomal replication protein N'' (priC) |
|  |  | K02317 | DNA replication protein DnaT (dnaT) |
| **Non-homologous end-joining** | **End binding and processing** | K10979 | DNA end-binding protein Ku (ku) |
|  | **Gap filling and ligation** | K01971 | bifunctional non-homologous end joining protein LigD (ligD) |
| **Antioxidation** | **Antioxidation** | K04564 | superoxide dismutase, Fe-Mn family (SOD2) |
|  |  | K04565 | superoxide dismutase, Cu-Zn family (SOD1) |
|  |  | K03781 | Catalase (CAT) |
|  |  | K03782 | catalase-peroxidase (katG) |

**Table S3. The marker genes for each conversion step of cold, acid and osmotic adaption in the Union Glacier area.**

|  | **Adaption mechanism** | **KEGG** | **Gene (Marker gene)** |
| --- | --- | --- | --- |
| **Cold adaption** | **Cold-shock protein** | K03704 | cold shock protein (beta-ribbon, CspA family) (cspA) |
|  | **Cold-adapted enzyme** | K00259 | cold-adapted alanine dehydrogenase (ald) |
|  | **Antifreeze protein** | K08305 | antifreeze glycopeptide (AFGP) |
|  | **Biosynthesis of unsaturated fatty acid** (K00059+ K00232+ K00507+ K01782+ K01825+ K03921+ K07513+ K10258) | K00059 | 3-oxoacyl-[acyl-carrier protein] reductase (fabG) |
|  |  | K00232 | acyl-CoA oxidase (ACOX1/ACOX3) |
|  |  | K00507 | stearoyl-CoA desaturase (Delta-9 desaturase) (SCD/desC) |
|  |  | K01782 | 3-hydroxybutyryl-CoA epimerase (fadJ) |
|  |  | K01825 | enoyl-CoA isomerase (fadB) |
|  |  | K03921 | acyl-[acyl-carrier-protein] desaturase (FAB2/SSI2/desA1) |
|  |  | K07513 | acetyl-CoA acyltransferase 1 (ACAA1) |
|  |  | K10258 | very-long-chain enoyl-CoA reductase (TER/TSC13/CER10) |
| **Acid adaption** | **Acid resistant protein** K19777+K14205 | K19777 | acid stress chaperone HdeA (hdeA) |
|  |  | K14205 | transmembrane acid tolerance protein |
| **Osmotic adaption** | **Osmotically inducible protein** K04062+K04063+K04064  +K04065 | K04065 | hyperosmotically inducible periplasmic protein (OsmY) |
|  |  | K04064 | osmotically inducible lipoprotein (OsmE) |
|  |  | K04063 | osmotically inducible protein (OsmC) |
|  |  | K04062 | osmotically inducible lipoprotein (OsmB) |

**Table S4.** **Percentages of genera present in all samples in the Union Glacier area.**

|  | CP14 | EH2 | EH5 | EH7 | GU7 | GU8 |
| --- | --- | --- | --- | --- | --- | --- |
| *Halomonas* | 6.11 | 68.71 | 32.02 | 56.35 | 19.04 | 19.74 |
| *Sphingomonas* | 17.31 | 1.13 | 2.20 | 3.41 | 3.55 | 0.72 |
| *Escherichia* | 3.10 | 3.78 | 7.57 | 0.22 | 0.74 | 7.22 |
| *Nesterenkonia* | 0.28 | 4.74 | 2.50 | 4.50 | 1.35 | 1.87 |
| *Pelagibacterium* | 0.33 | 3.55 | 1.59 | 3.18 | 0.83 | 0.96 |
| *Pseudomonas* | 0.16 | 0.67 | 2.90 | 1.20 | 0.48 | 0.24 |
| *Actinotalea* | 0.49 | 0.52 | 0.91 | 0.38 | 1.75 | 1.53 |
| *Aminobacter* | 1.37 | 0.74 | 0.25 | 0.44 | 0.16 | 0.36 |
| *Hoeflea* | 0.38 | 1.70 | 1.04 | 1.66 | 0.45 | 0.48 |
| *Dolosigranulum* | 0.19 | 1.38 | 0.43 | 0.70 | 0.45 | 0.48 |

**Table S5.** **Percentages of taxonomic population for acid and osmotic adaption in the Union Glacier area.**

| Phylum | Genus | Osmotic adaption | Acid adaption |
| --- | --- | --- | --- |
| Alphaproteobacteria | *Sphingomonas* | 0.00 | 0.54 |
| Alphaproteobacteria | *Sphingobium* | 0.00 | 11.05 |
| Alphaproteobacteria | *Rhodobacter* | 11.13 | 0.00 |
| Alphaproteobacteria | *Rhizorhabdus* | 3.45 | 0.00 |
| Alphaproteobacteria | *Niveispirillum* | 0.00 | 1.04 |
| Alphaproteobacteria | *Mesorhizobium* | 1.08 | 0.00 |
| Alphaproteobacteria | *Chelatococcus* | 5.17 | 0.00 |
| Alphaproteobacteria | *Caulobacter* | 0.00 | 3.89 |
| Alphaproteobacteria | *Brucella* | 0.00 | 31.31 |
| Alphaproteobacteria | *Bradyrhizobium* | 1.78 | 0.00 |
| Betaproteobacteria | *Ralstonia* | 1.27 | 1.00 |
| Betaproteobacteria | *Orrella* | 1.54 | 0.00 |
| Betaproteobacteria | *Methylotenera* | 1.37 | 0.00 |
| Betaproteobacteria | *Laribacter* | 0.00 | 34.60 |
| Betaproteobacteria | *Bordetella* | 1.93 | 0.00 |
| Deltaproteobacteria | *Vulgatibacter* | 1.44 | 0.00 |
| Deltaproteobacteria | *Desulfococcus* | 6.18 | 0.00 |
| Gammaproteobacteria | *Rahnella* | 1.11 | 0.00 |
| Gammaproteobacteria | *Pseudoxanthomonas* | 1.22 | 0.00 |
| Gammaproteobacteria | *Lysobacter* | 1.71 | 0.00 |
| Gammaproteobacteria | *Leclercia* | 3.44 | 0.00 |
| Gammaproteobacteria | *Escherichia* | 1.23 | 0.00 |
| Gammaproteobacteria | *Enterobacteriaceae bacterium strain FGI 57* | 4.27 | 0.00 |
| Gammaproteobacteria | *Shigella* | 0.00 | 1.58 |
| Gammaproteobacteria | *Pseudomonas* | 13.60 | 3.46 |
| Gammaproteobacteria | *Azotobacter* | 0.00 | 7.02 |
| Actinobacteria | *Thermobispora* | 1.38 | 0.00 |
| Actinobacteria | *Rubrobacter* | 2.24 | 0.00 |
| Acidobacteria | *Luteitalea* | 6.89 | 0.00 |
| Firmicutes | *Lysinibacillus* | 0.00 | 1.53 |
| Firmicutes | *Bacillus* | 0.00 | 1.37 |
| Bacteroidetes | *Cyclobacterium* | 3.81 | 0.00 |
| Bacteroidetes | *Algoriphagus* | 1.46 | 0.00 |
| Cyanobacteria | *Stanieria* | 1.05 | 0.00 |
| Unclassified Terrabacteria group | *Thermobaculum* | 8.42 | 0.00 |
| Others | Others | 12.25 | 1.61 |

**Table S6. The percentage of each conversion step for carbon, nitrogen and sulfur cycles in the Union Glacier area.**

|  | **Pathway** | **Percent (%)** |
| --- | --- | --- |
| **Sulfur** | Assimilatory sulfate reduction | 32.50 |
|  | Dissimilatory sulfate reduction and sulfide oxidation | 0.03 |
|  | Sulfur mineralization | 67.42 |
|  | Polysulfide reduction | 0.05 |
| **Carbon** | Aerobic C fixation | 5.44 |
|  | Aerobic methane oxidation | 2.01 |
|  | Aerobic respiration | 52.83 |
|  | Anaerobic C fixation | 13.00 |
|  | CO oxidation | 19.93 |
|  | Fermentation | 6.66 |
|  | Methanogenesis | 0.13 |
| **Nitrogen** | Ammonification | 9.38 |
|  | Denitrification | 9.42 |
|  | Nitrate reduction + Nitrite oxidation | 0.89 |
|  | Nitrate reduction | 1.36 |
|  | Nitrification | 0.93 |
|  | Nitrogen assimilation | 64.93 |
|  | Nitrogen fixation | 0.41 |
|  | Nitrogen mineralization | 12.68 |
